# Urinary Post-Translationally Modified Fetuin-A (uPTM-FetA) in Chronic Kidney Disease Patients with and without Diabetic Kidney Disease

**DOI:** 10.3390/medicina60030363

**Published:** 2024-02-21

**Authors:** Michela Musolino, Marta Greco, Mario D’Agostino, Loredana Tripodi, Roberta Misiti, Francesco Dragone, Paola Cianfrone, Mariateresa Zicarelli, Daniela Patrizia Foti, Michele Andreucci, Davide Bolignano, Giuseppe Coppolino

**Affiliations:** 1 Nephrology and Dialysis Unit, Magna Graecia University Hospital, 88100 Catanzaro, Italy; 2 Department of Health Sciences, Magna Graecia University, 88100 Catanzaro, Italy; 3 Clinical Pathology Lab., Magna Graecia University Hospital, 88100 Catanzaro, Italy; 4 Department of Medical and Surgical Sciences, Magna Graecia University, 88100 Catanzaro, Italy

**Keywords:** post-translational modified form of human fetuin-A, chronic kidney disease, diabetic kidney disease, biomarker

## Abstract

*Background and Objectives:* A novel post-translational modification (PTM) fragment derived from the cleavage of Fetuin-A (PTM-FetA) has recently emerged as a sensitive biomarker for kidney damage in diabetic patients, but evidence in other chronic renal diseases is lacking. In this pilot study, we aimed at evaluating the clinical significance of urinary PTM-FetA (uPTM-FetA) in a mixed cohort of patients with non-advanced chronic kidney disease (CKD) secondary to diabetic kidney disease (DKD) or other causes. *Materials and Methods:* We enrolled 47 adult patients with CKD (mean CKD-Epi 40.10 ± 16.5 mL/min/1.73 m^2^) due to DKD (*n* = 34) or other etiology (*n* = 13). uPTM-FetA was measured in the urine using a commercially available ELISA kit. Fifteen healthy individuals served as controls. *Results:* Collectively, all CKD patients displayed remarkably higher levels of uPTM-FetA than controls (0.84 [0.10–1.15] vs. 29.68 [2.50–55.16] ng/mL *p* = 0.0005), but values were lower in non-DKD than in DKD patients (1.66 [0.09–4.19] vs. 13.9 [0.01–45.02] ng/mL; *p* = 0.01). uPTM-FetA showed a great diagnostic capacity at ROC analyses to identify the presence of CKD (AUC 0.776; *p* < 0.001) and, within CKD patients, to discriminate the diabetic and non-diabetic etiology (AUC 0.673; *p* = 0.02). At multivariate correlation analyses, proteinuria (β = 0.442; *p* = 0.02) and BMI (β = −0.334; *p* = 0.04) were the sole independent predictors of uPTM-FetA in this study population. *Conclusions:* uPTM-FetA could be a novel sensitive biomarker at the crossroad of chronic renal damage and metabolic dysfunction. Additionally, this biomarker could also represent a non-invasive, complementary tool for discriminating among different CKD etiologies (DKD vs. non-DKD) in difficult cases or when renal biopsy is not available.

## 1. Introduction

Chronic kidney disease (CKD) is nowadays considered a true health priority, given the impressive rise in its incidence over recent decades and the consequent heavy impact on healthcare costs worldwide [1,2,3]. Given the paucity of clinical manifestations in early stages, a late identification of this condition is not infrequent, and may delay the timely establishment of therapeutic measures. On the other hand, misdiagnosis regarding the true CKD etiology may lead to inappropriate interventions [4,5]. As an example, despite diabetic kidney disease (DKD) representing the leading cause of CKD worldwide [6], diabetic patients with other concomitant renal disorders, such as glomerulonephritis, are often erroneously labeled as suffering DKD [7].

Indeed, albuminuria and proteinuria, which remain the cornerstone for the diagnosis, follow-up and therapeutic management of DKD, may display questionable sensitivity and specificity, as they may increase due to other glomerular disorders or may remain within the normal range in up to 40% of diabetic patients with biopsy evidence of renal involvement [8]. On the other hand, renal histology is invasive, time-consuming, and often not feasible or not recommended in diabetic patients. The search for alternative or complementary biomarkers for the diagnostic and clinical stratification of CKD and DKD patients, thus remains a timely research priority.

In this pilot, cross-sectional study, we explored the possible clinical significance of measuring urinary levels of the post-translational modified form of human fetuin-A (PTM-FetA) in a mixed cohort of patients with non-advanced CKD of various etiology. PTM-FetA is a new, emerging urinary biomarker which has recently been investigated for the prediction of the progression of renal impairment in type 2 diabetic patients [9].

In our study, we hypothesized that altered uPTM-FetA levels may reflect chronic kidney damage and may hold usefulness for the diagnostic stratification of individuals with CKD.

## 2. Materials and Methods

### 2.1. Study Cohort

Participants to this study were enrolled drawing on an outpatient population already followed at the Nephrology Unit of the University Hospital of “Magna Graecia” of Catanzaro, Italy. The main inclusion criteria were the presence of a mild-to-moderate renal failure (CKD stages 2–4, eGFR between 89 and 15 mL/min/1.73 m^2^ estimated by the CKD-Epi formula) and a stable renal function, defined as the absence of a >25% decline in eGFR during the 6 months prior to the start of the study. Exclusion criteria were the following: presence of ongoing infections, cancer, recent cardiovascular events requiring hospitalization, inflammatory states, uncontrolled hypertension, severe proteinuria (>3 g/24 h), and previous kidney transplant. Additionally, 15 healthy matched individuals with no history of kidney disease or concomitant active diseases served as controls for uPTM-FetA measurement. The study was approved by the local ethics committee and all participants signed an informed consent.

### 2.2. Clinical Assessment

Clinical, demographic, laboratory and anthropometric parameters were recorded during a planned outpatient visit, and were entered into an electronic standardized report form, together with information on active therapy. The recent clinical history of the participants was reconstructed through anamnesis in the context of clinical examination and consultation of the medical records. The identification of CKD etiology was available in all patients, and was based on clinical history (nephroangiosclerosis, chronic pyelonephritis), genetics (ADPKD) or renal biopsy (glomerulonephritis, DKD). Additionally, blood pressure was measured upon the planned visit three times using a sphygmomanometer, and its average value considered for data analysis.

### 2.3. Post-Translationally Modified Fetuin-A Fragments in Urine (uPTM-FetA) Measurement

uPTM-FetA was measured in early morning spot urine samples before the planned outpatient visit using a commercial ELISA kit (Human uPTM3-DKD ELISA; Bio Preventive Medicine Corp, Zubei City, Taiwan; Trade name: DNlite-IVD103 distributed by Tecan, Cernusco sul Naviglio, Italy). In this assay, calibrators or urine samples were mixed with anti-unique PTM Fetuin-A monoclonal antibody (mAb), and then incubated in a microplate pre-bounded with unique PTM Fetuin-A. After an incubation, a Horse Radish Peroxide (HRP) conjugated secondary antibody was added, followed by an incubation with 3,3′,5,5′-tetramethylbenzidine (TMB) substrate, according to the manufacturer’s instructions. Their relative reactivity was determined by absorbance measurement at 450 nanometers (nm) and plotted by comparison with a predetermined unique PTM Fetuin-A calibration curve. The linearity of Human uPTM3-DKD ELISA kit was found to be from 5.38–454.20 ng/mL (R^2^ = 0.99) with a limit of detection being 1.58% ng/mL. The precision has been validated by measuring two samples in triplicate in 2 lots for 20 days (CV = 12%); the reproducibility has been validated by measuring two samples in 5 replicate in 3 sites for 4 days (CV = 18%). uPTM-FetA values were normalized for urine volume and expressed as ng/mL.

### 2.4. Statistical Analysis

The analyses were performed using the SPSS package (version 24.0; IBM corporation, Armonk NY, USA) and the MedCalc Statistical Software (version 14.8.1, MedCalc Software bvba, Ostend, Belgium). Data were presented as mean  ±  SD for normally distributed values (via Kolmogorov–Smirnov test), median [IQ range] for variables with skewed distribution, or frequency percentage, as appropriate. Differences between groups were determined by the unpaired *T*-test for normally distributed values, the Mann–Whitney U test for non-parametric values, and the chi-square followed by a Fisher’s exact test for frequency distributions. The Pearson (R) and the Spearman (Rho) correlation coefficients were employed to identify the univariate correlates of uPTM-FetA. Multiple regression analyses were thus performed to assess independent relationships, considering uPTM-FetA as the dependent variable. Receiver Operating Characteristics (ROC) analyses were employed to calculate the area under the curve (AUC) for uPTM-FetA, considering the presence of CKD in the complete study population (CKD patients + healthy controls) and that of DKD in the CKD population as the status variable, respectively. The best cut-off values for uPTM-FetA were computed by the Youden index. All results were considered significant if the *p* value was <0.05.

## 3. Results

### 3.1. Clinical Characteristics and uPTM-FetA Measurement in the Study Cohort

The study population consisted of 47 adult patients (mean age 71.6 ± 10 years, 63.8% male) who met the inclusion/exclusion criteria for being eligible to participate. DKD was the established cause of CKD in 34 patients (72.3%), while in the remaining 13 individuals CKD was related to glomerulonephritis (*n* = 5), ADPKD (*n* = 2), chronic pyelonephritis (*n* = 2) or nephroangiosclerosis (*n* = 4). Mean eGFR (CKD-Epi) was 40.10 ± 16.5 mL/min/1.73 m^2^ in the whole study population, being marginally higher (*p* = 0.05) in the DKD (38.5 ± 12.6 mL/min/1.73 m^2^) compared to the non-DKD group (43.3 ± 9.8 mL/min/1.73 m^2^). BMI was significantly higher in the DKD vs. non-DKD group (28.5 ± 5.1 vs. 25.6 ± 5.1 *p* = 0.01), as well as proteinuria (0.381 [0.080–0.751] vs. 0.14 [0.168–0.312] *p* = 0.04), while the systolic blood pressure was lower (133.6 ± 18.8 mmHg vs. 125.4 ± 15.6 mmHg *p* = 0.05).

DKD patients were more frequently under SGLT-2 inhibitors treatment (50% vs. 23%; *p* = 0.03) and statins (97% vs.46.1%; *p* = 0.01), while no differences were observed for other therapeutics.

**Figure 1:**
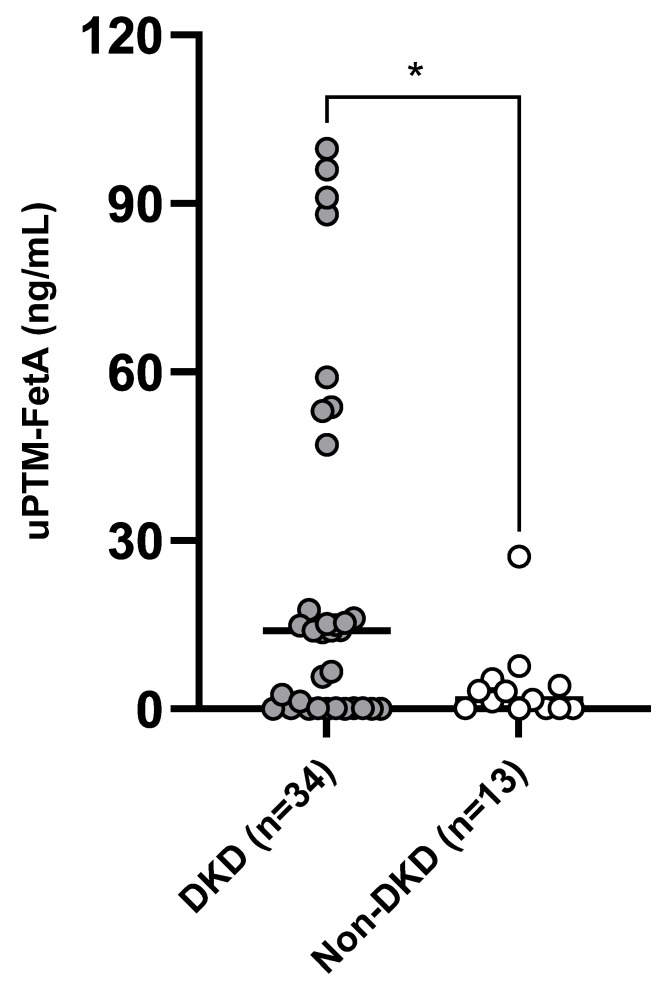
Difference in median uPTM-FetA levels between DKD and non-DKD patients. * *p* = 0.01.

uPTM-FetA values were remarkably higher in the whole study population as compared with the control group (29.68 [2.50–55.16] vs. 0.84 [0.10–1.15] ng/mL; *p* = 0.0005). In parallel, a significant difference was also found in uPTM-FetA values between DKD and Non-DKD individuals (13.9 [0.01–45.02] vs. 1.66 [0.09–4.19]; *p* = 0.01; Figure 1). Such observations were confirmed at exploratory analyses (not shown) employing uPTM-FetA normalized by urinary creatinine.

Table 1 summarizes the complete information regarding the characteristics and active therapy for all the CKD patients and for the two subgroups (DKD and non-DKD).

### 3.2. Clinical Correlates of uPTM-FetA in CKD Patients

At univariate analyses, uPTM-FetA was negatively associated with BMI (R = −0.323; *p* = 0.02), Vitamin-D (R = −0.517; *p* = 0.005) and directly with proteinuria (R = 0.312; *p* = 0.04), gammaGT (R = 0.775 *p* = 0.02) and urinary potassium (R = 0.382 *p* = 0.04). In a fully adjusted multivariate model including all the univariate correlates, only proteinuria (β = 0.442; *p* = 0.02) and BMI (β = −0.334; *p* = 0.04) remained significantly associated with uPTM-FetA, while the other associations were lost. Of note, this resulting model was remarkably robust, as it was able to explain a large percentage of the uPTM-FetA variability in this population (Multiple R = 0.91, R^2^ = 83%; *p* = 0.007). Table 2 summarizes results from univariate and multivariate analysis of uPTM-FetA.

### 3.3. ROC Analyses of uPTM-FetA

**Figure 2:**
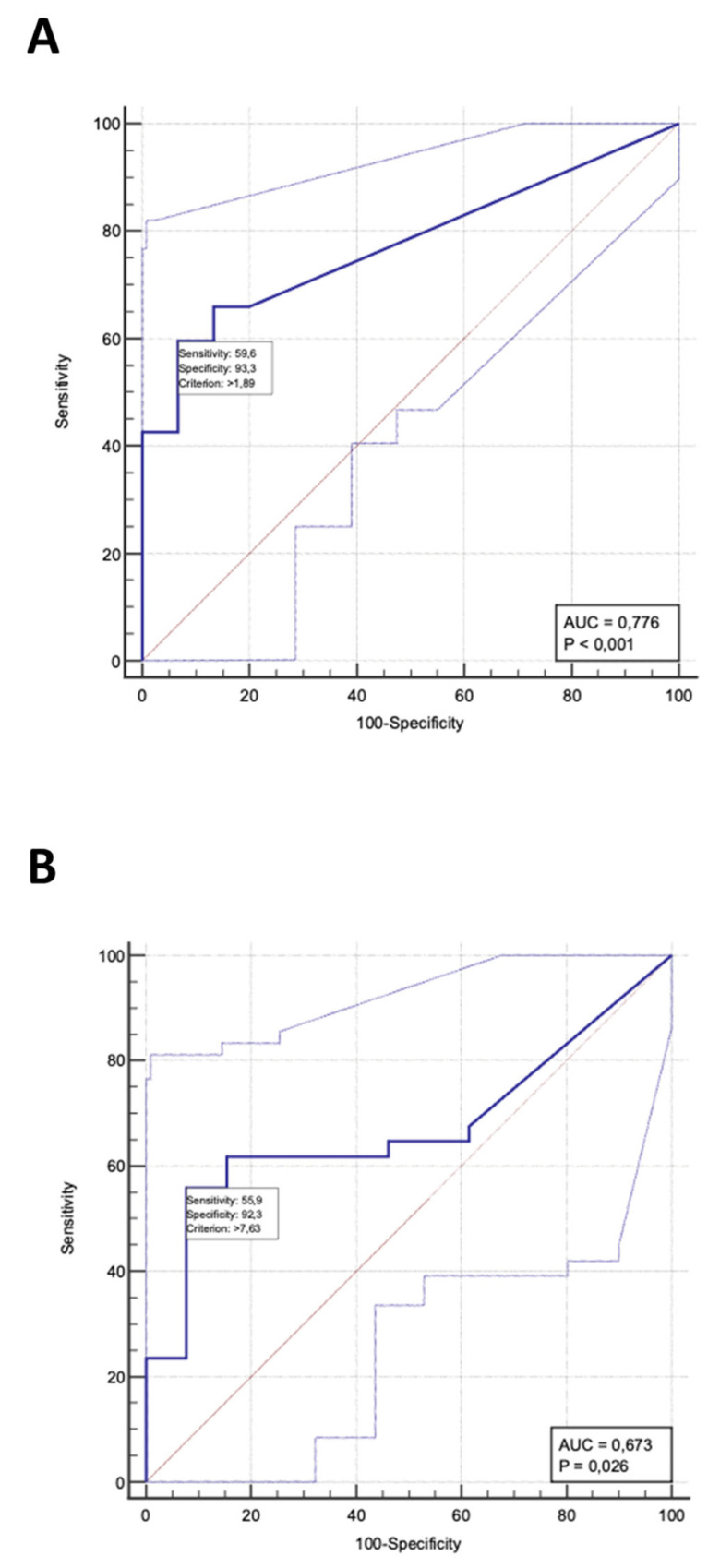
ROC curves of uPTM-FetA in discriminating (**A**) the presence of CKD in the whole study cohort (patients + controls) and (**B**) DKD as the etiology of CKD.

At ROC analyses, uPTM-FetA showed a remarkable capacity to discriminate CKD patients from healthy individuals with an area under the curve (AUC) of 0.776 (*p* < 0.001) and a best cut-off value of 1.89 ng/mL (sensitivity 59.6% 95%CI 44.3–73.6; specificity 93.3%, 95%CI 68.1–99.8). By the same token, within all CKD individuals, uPTM-FetA displayed a good diagnostic ability in identifying DKD as the etiology of CKD with an AUC 0.673 (*p* = 0.02) and a best cut-off value of 7.63 ng/mL (sensitivity 55.9% 95%CI 37.9–72.8; specificity 92.3%, 95%CI 64–99.8) (Figure 2).

## 4. Discussion

In accordance with Chuang et al. [9], in this pilot study, we confirmed the ability of uPTM-FetA to reflect, with a good sensitivity and specificity, the presence of a chronic impairment in renal function. However, unlike the Chuang study, which focused specifically on diabetic individuals, we demonstrated that the utility of this biomarker could go beyond the sole DKD setting. In fact, in our mixed CKD cohort, DKD individuals showed significantly higher levels of uPTM-FetA with respect to individuals with chronic renal damage due to other etiology. Starting from these premises, we were able to uncover a satisfactory diagnostic capacity of this biomarker at ROC analyses in discriminating between different causes of CKD (DKD vs. non-DKD). Hence, the measurement of uPTM-FetA could help not only CKD identification, but also its etiological stratification, particularly in individuals with more advanced CKD stages or when renal biopsy is not feasible or available.

Fetuin-A, which is structured in two chains (A and B), becomes active after a proteolytic division of these two parts [10]. Magalhaes et al. recently identified 14 different urinary protein fragments belonging to the region of the connecting peptide of the total fetuin-A protein which are significantly increased in the urine of type 2 diabetic patients with already manifested kidney disease [11].

Post-translational modifications (PTMs) can play a crucial role in regulating the structure and function of proteins, impacting various cellular processes and intracellular signaling pathways. In a large proteomic profiling of urines from the participants to the Taiwan Renal Biomarker Study, uPTM-FetA emerged as one of the best biomarker candidates for the identification of DKD in type 2 diabetes [9]. Conversely, its usefulness in other CKD conditions remained, to date, unexplored.

Fetuin-A, is primarily synthesized in the liver and secreted into the bloodstream, playing a role in various physiological processes, including calcium homeostasis, inhibition of soft tissue calcification, and regulation of insulin sensitivity [12]. Beyond these roles, this protein is crucially involved in the pathogenesis of inflammation, insulin resistance and obesity [13].

Accordingly, in our cohort, we found BMI as the sole significant independent predictor of uPTM-FetA levels, together with proteinuria which, in turn, reflects the severity of active renal damage in the context of chronic kidney impairment. Taken all together, these assumptions could thus place uPTM-FetA at the crossroad of metabolic dysfunction and kidney damage, but targeted mechanistic studies are needed to confirm this hypothesis.

Indeed, the mechanisms underlying the increased urinary fetuin-A levels, during renal damage, still remain poorly understood. In the presence of kidney injury, fetuin-A could be lost, with a higher amount, in urine through the damaged glomerulus or, alternatively, could be actively synthetized and also released in urine from renal cells as part of a local protective reaction [14]. Accordingly, Hua Zhou et al. found very high concentrations of exosomal Fetuin-A during acute kidney injury [15], and variations in its levels of this biomarker overtime may reflect an accelerated progression of particular renal disorders like autosomal polycystic kidney disease [16]. In this last regard, the lack of a prospective phase in our pilot study prevents us to assess the utility of uPTM-FetA as a predictor of progression in such a mixed CKD, as previously demonstrated in DKD patients only [9]. By the same token, our preliminary findings, despite promising, deserve appropriate validation in larger and more heterogeneous cohorts and, ideally, should be accompanied by more in-depth sensitive and subgroup analyses taking into account, for instance, the different sub-etiologies of CKD in individuals without DKD.

## 5. Conclusions

uPTM-FetA could be a novel sensitive biomarker reflecting renal damage in CKD patients with or without DKD. In this regard, this protein could represent an additive tool for discriminating the diabetic etiology of CKD from other causes, particularly in difficult cases or where biopsy is not available. Further studies are needed to evaluate whether uPTM-FetA measurement may predict different disease evolution in CKD patients.

## Figures and Tables

**Table 1:** Main clinical characteristics in the whole study cohort and in patients categorized according to CKD etiology (DKD vs. others). Statistically significant differences between subgroups are highlighted in bold.

	All CKD *n*: 47	Non-DKD *n*: 13	DKD *n*: 34	*p*
Age (years)	71.6 ± 10	72 ± 8.8	70.9 ± 9.5	0.32
Male sex (%)	63.8	61.5	64.7	0.77
Anti-hypertensive therapy				
-ACEi/ARBs (%)	85.1	84.6	90.6	0.22
-Beta-blockers (%)	44.6	38.4	47	0.56
-CCBs (%)	44.6	38.4	47	0.56
-Aldosterone antagonists (%)	6.4	7.6	5.9	0.68
-Furosemide (%)	80.8	76.9	82.3	0.35
**SGLT-2 inhibitors (%)**	**42.5**	**23**	**50**	**0.03**
**Statins (%)**	**82.9**	**46.1**	**97**	**0.01**
**BMI (Kg/m^2^)**	**28.7 ± 5.3**	**25.6 ± 5.1**	**28.5 ± 5.1**	**0.01**
**Systolic BP (mmHg)**	**130 ± 18.2**	**133.6 ± 18.8**	**125.4 ± 15.6**	**0.05**
Diastolic BP (mmHg)	72.5 ± 9.3	71 ± 8.2	73.2 ± 11.8	0.37
**CKD-Epi eGFR (mL/min/1.73 m^2^)**	**40.1 ± 16.5**	**38.5 ± 12.6**	**43.3 ± 9.8**	**0.05**
**Serum Creatinine (mg/dL)**	**1.8 ± 0.66**	**1.95 ± 0.77**	**1.68 ± 0.45**	**0.05**
**Urea (mg/dL)**	**78.9 ± 27.1**	**83.8 ± 29.6**	**77.8 ± 19.6**	**0.05**
Albumin (g/dL)	4.28 ± 0.44	4.31 ± 0.65	4.19 ± 0.28	0.68
Serum Sodium (mmol/L)	140.2 ± 3.6	138.7 ± 38	141.2 ± 2.3	0.38
Serum Potassium (mmol/L)	4.25 ± 0.31	4.54 ± 0.61	4.13 ± 0.35	0.55
Serum Calcium (mg/dL)	9.02 ± 0.13	9 ± 0.81	9.12 ± 0.39	0.88
Serum Phosphate (mg/dL)	3.8 ± 0.83	3.82 ± 0.54	3.76 ± 0.36	0.54
Red blood cells (n × 10^6^)	4.5 ± 0.9	4.45 ± 0.9	4.69 ± 0.6	0.88
Hemoglobin (g/dL)	12.5 ± 0.7	12.4 ± 0.5	12.5 ± 1.6	0.26
**Platelets (n × 10^3^)**	**228.8 ± 54**	**235 ± 65.7**	**200.6 ± 70.1**	**0.05**
**Total Cholesterol (mg/dL)**	**146.3 ± 38.6**	**139.5 ± 16.2**	**172.6 ± 18.2**	**0.04**
LDL Cholesterol (mg/dL)	74 ± 18	70 ± 21.7	75 ± 18.7	0.11
Triglycerides (mg/dL)	126[81–173.2]	140.1 [77.1–180]	119 [3.2–99.8]	0.61
iPTH (pg/mL)	84.3 [42.7–165.3]	103 [44.8–201.4]	79 [51.7–133]	0.08
Uric Acid (mg/dL)	5.2 ± 1.2	5.32 ± 1.41	5.1 ± 1.64	0.90
Creatinuria (mg/dL)	11.5 [9.4–16]	11.1 [3.1–13.2]	11.4 [9.4–17.2]	0.37
**Proteinuria (g/24 h/1.73 m^2^)**	**0.266 [0.124–0.667]**	**0.14 [0.168–0.312]**	**0.381 [0.080–0.751]**	**0.04**
Urine sodium (mg/24 h)	150.7 ± 58.8	153.3 ± 59.9	149.6 ± 40.9	0.56
Urine potassium (mg/24 h)	49.2 ± 11.3	50.2 ± 9.1	49.1 ± 11.8	0.77
**uPTM-FetA (ng/mL)**	**29.68 [2.50–55.16]**	**1.66 [0.09–4.19]**	**13.9 [0.01–45.02]**	**0.01**

BMI: body mass index, BP: blood pressure, CCBs: calcium channel blockers, CKD-Epi eGFR: estimated glomerular filtration rate by the CKD-Epi formula, iPTH: intact Parathormone, uPTM-FetA: urinary post-translational modified form of human fetuin-A.

**Table 2:** Univariate and multiple regression analysis of (log-transformed) uPTM-FetA levels. Statistically significant correlations are highlighted in bold.

	R	*p*
**BMI**	**−0.323**	***p* = 0.02**
**Vitamin-D**	**−0.517**	***p* = 0.005**
**Proteinuria**	**0.312**	***p* = 0.04**
**Gamma-GT**	**0.775**	***p* = 0.02**
**Urinary Potassium**	**0.382**	***p* = 0.04**
	**β**	***p***
**BMI**	**−0.334**	***p* = 0.04**
**Proteinuria**	**0.442**	***p* = 0.02**
Vitamin-D	0.097	*p* = 0.35
Gamma-GT	0.123	*p* = 0.45
Urinary Potassium	−0.012	*p* = 0.63

Multiple R = 0.91, R^2^ = 83%; *p* = 0.007.

## Data Availability

Raw data are available from the Corresponding Author upon reasonable request.
